# Simultaneous Detection of CDC Category “A” DNA and RNA Bioterrorism Agents by Use of Multiplex PCR & RT-PCR Enzyme Hybridization Assays

**DOI:** 10.3390/v1030441

**Published:** 2009-10-20

**Authors:** Jie He, Andrea J. Kraft, Jiang Fan, Meredith Van Dyke, Lihua Wang, Michael E. Bose, Marilyn Khanna, Jacob A. Metallo, Kelly J. Henrickson

**Affiliations:** 1 Department of Pediatric, Medical College of Wisconsin, Milwaukee, WI 53226, USA; E-Mails: jhe@mcw.edu (J.H.); akraft@mcw.edu (A.J.K.); jfan@mcw.edu (J.F.); mvandyke@mcw.edu (M.V.D.); lwang@mcw.edu (L.W.); mbose@mcw.edu (M.E.B.); jmetallo@mcw.edu (J.A.M.); 2 National Institutes of Health, Bethesda, MD 20892-7616, USA; E-Mail: khannama@niaid.nih.gov (M.K.); 3 Children’s Research Institute, Children’s Hospital of Wisconsin, P.O. Box 1997, Milwaukee, WI 53201-1997, USA; 4 Children’s Hospital of Wisconsin, P.O. Box 1997, Milwaukee, WI 53201-1997, USA

**Keywords:** Multiplex PCR, simultaneous detection, *Variola major*, *Bacillus anthracis*, *Yersinia pestis*, *Francisella tularensis*, Ebola virus, Lassa fever virus, Rift Valley Fever virus, Hantavirus, dengue virus

## Abstract

Assays to simultaneously detect multiple potential agents of bioterrorism are limited. Two multiplex PCR and RT-PCR enzyme hybridization assays (mPCR-EHA, mRT-PCR-EHA) were developed to simultaneously detect many of the CDC category “A” bioterrorism agents. The “Bio T” DNA assay was developed to detect: *Variola major* (VM), *Bacillus anthracis* (BA), *Yersinia pestis* (YP), *Francisella tularensis* (FT) and Varicella zoster virus (VZV). The “Bio T” RNA assay (mRT-PCR-EHA) was developed to detect: Ebola virus (Ebola), Lassa fever virus (Lassa), Rift Valley fever (RVF), Hantavirus Sin Nombre species (HSN) and dengue virus (serotypes 1–4). Sensitivity and specificity of the 2 assays were tested by using genomic DNA, recombinant plasmid positive controls, RNA transcripts controls, surrogate (spiked) clinical samples and common respiratory pathogens. The analytical sensitivity (limit of detection (LOD)) of the DNA asssay for genomic DNA was 1×10^0^∼1×10^2^ copies/mL for BA, FT and YP. The LOD for VZV whole organism was 1×10^−2^ TCID_50_/mL. The LOD for recombinant controls ranged from 1×10^2^∼1×10^3^copies/mL for BA, FT, YP and VM. The RNA assay demonstrated LOD for RNA transcript controls of 1×10^4^∼1×10^6^ copies/mL without extraction and 1×10^5^∼1×10^6^ copies/mL with extraction for Ebola, RVF, Lassa and HSN. The LOD for dengue whole organisms was ∼1×10^−4^ dilution for dengue 1 and 2, 1×10^4^ LD_50_/mL and 1×10^2^ LD_50_/mL for dengue 3 and 4. The LOD without extraction for recombinant plasmid DNA controls was ∼1×10^3^ copies/mL (1.5 input copies/reaction) for Ebola, RVF, Lassa and HSN. No cross-reactivity of primers and probes used in both assays was detected with common respiratory pathogens or between targeted analytes. Clinical sensitivity was estimated using 264 surrogate clinical samples tested with the BioT DNA assay and 549 samples tested with the BioT RNA assay. The clinical specificity is 99.6% and 99.8% for BioT DNA assay and BioT RNA assay, respectively. The surrogate sensitivities of these two assays were 100% (95%CI 83–100) for FT, BA (pX02), YP, VM, VZV, dengue 2,3,4 and 95% (95%CI 75–100) for BA (pX01) and dengue 1 using spiked clinical specimens. The specificity of both BioT multiplex assays on spiked specimens was 100% (95% CI 99–100). Compared to other available assays (culture, serology, PCR, *etc*.) both the BioT DNA mPCR-EHA and BioT RNA mRT-PCR-EHA are rapid, sensitive and specific assays for detecting many category “A” Bioterrorism agents using a standard thermocycler.

## Introduction

Biological weapons pose a significant threat. Many virulent and pathogenic organisms can be used individually or together as weapons. This use could cause not only high rates of morbidity and mortality among the population but also significant societal disruption and cost [1,2]. The Centers for Disease Control and Prevention classified these biological agents as category “A”, “B” or “C” agents based on the potential of threat. The category “A” bioterrorism agents include *Variola major* (VM, smallpox), *Bacillus anthracis* (BA, anthrax), *Yersinia pestis* (YP, plague), *Clostridium botulinum* (botulism), *Francisella tularensis* (FT, tularemia) and a group of RNA viruses that cause hemorrhagic fevers (VHFs) [3–8]: Lassa fever virus and New World arenaviruses (*Arenaviridae*), Rift Valley fever (RVF) and hantavirus (*Bunyaviridae*), Ebola virus and Marburg viruses (*Filoviridae*) and dengue virus, Omsk hemorrhagic fever virus and Kyanasur Forest disease virus (*Flaviviridae*).

In a suspected bioterrorist attack, rapid diagnosis is critical for effective public health responses. The treatment for the various DNA organisms is very specific and would be possible only if the causative organisms were promptly identified. All of the described RNA viruses except for dengue are highly infectious by aerosol transmission and no specific treatment has been demonstrated as safe and effective for the hemorrhagic fevers [7]. The rapid screening of suspected samples would help control the spread and ensure accurate treatment of the diseases. Current available diagnostic tests for CDC category “A” bioterrorism agents (e.g., cell culture, serology) are time consuming, have less than optimal sensitivity and specificity or cannot test for multiple agents simultaneously [9–12]. Newer molecular methods are either singleplex real-time PCR assays that are not available outside the CDCs LRN network or small commercial multiplex PCR assays that are linked to expensive machines (and also not easily available) [13,14]. There is a need for precise, rapid, simple and robust diagnostic assays to detect a large number of these agents for both clinical treatment and to prevent panic among the public and authorities.

Large multiplex PCR assays have the advantage of detecting many pathogens from the same clinical sample in only a few hours while being highly sensitive and specific [15–19]. In this present study, two large multiplex-PCR assays were developed for simultaneous detection of many of the CDC category “A” DNA and RNA based bioterrorism agents. The BioT DNA multiplex PCR-enzyme hybridization assay (BioT DNA mPCR-EHA) was developed for simultaneous detection of: VM, BA, YP, and FT. The BioT RNA multiplex reverse transcription PCR-EHA (BioT RNA mRT-PCR-EHA) was developed for simultaneous detection of Ebola virus, Lassa virus, RVF virus, Hanta virus Sin Nombre species(HSN), and dengue virus (serotypes 1–4). Varicella-Zoster virus while not a category “A” agent was chosen as a target for the BioT DNA assay in order to positively identify the cause of rash or lesions in suspected smallpox patients.

## 2. Results

### 2.1. Analytical Sensitivity

Each assay’s analytical sensitivity was determined by testing duplicate DNA samples, RNA transcripts or whole organism for different analytes and repeated at least three times to determine the assay’s reproducibility. This LOD was determined by the lowest dilution which gave a positive EHA reading greater than 0.4. The LOD of the DNA assay for genomic DNA and whole organisms is shown in Table 1.

The LOD for genomic DNA was at 1×10^1^ copies/mL for BA pX01 and FT, 1×10^2^ copies/mL for BA pX02 and 1×10^0^ copies/mL for YP. The LOD for VZV whole organism was 1×10^−2^ TCID_50_/mL. The LOD for recombinant plasmid DNA controls was evaluated in the same way and is shown in Table 1. The LOD for both BA pX01 and BA pX02 recombinant controls was 1×10^2^ copies/mL. The analytical sensitivity for FT, YP and VM were 1×10^3^, 2.5×10^2^ and 5×10^2^ copies/mL, respectively.

The LOD of the RNA assay with or without extraction for RNA transcript control is shown in Table 2.

The LOD without extraction for RNA transcript control was at 1×10^6^ copies/mL for Ebola and RVF, 1×10^5^ copies/mL for Lassa and 1×10^4^ copies/mL for HSN. The LOD with extraction for RNA transcript control was at 1×10^5^ copies/mL for Ebola, Lassa and HSN, and 1×10^6^ copies/mL for RVF.

The LOD for dengue whole organism (4 serotypes) is shown in Table 2. The LOD for dengue whole organisms was approximately 1×10^−4^ dilution for dengue 1 and 2, 1×10^4^ LD_50_/mL for dengue 3 and 1×10^2^ LD_50_/mL for dengue 4.

The LOD without extraction for recombinant plasmid DNA controls was evaluated in the same way and is shown in Table 2. The LOD without extraction for recombinant plasmid DNA controls was at 1×10^3^ copies/mL for all 4 targets. This demonstrated outstanding sensitivity (∼1.5 input copies/reaction) for the primers and probes of the RNA assay in PCR-EHA and suggests that our RNA controls did not represent the LOD we would expect to see in true clinical testing. We hypothesize that our RNA transcripts are unstable and degradation is likely during the dilution and extraction process.

### 2.2. Analytical Specificity

The analytical specificity of the assays was determined using a number of closely related organisms and other common respiratory pathogens that might be found in clinical samples. These samples were tested for cross-reactivity to the primers and probes. Table 3 described the organisms, the dilutions used and the results.

No cross-reactivity between primers, probes and these organisms was detected in either assay. The internal control probe in the RNA assay can identify human parainfluenza virus 2 (HPIV-2) because the RNA assay used HPIV-2 as an internal control. HPIV-2 produced negative result for all other primers and probes in this assay except the internal control which should be positive.

### 2.3. Evaluation of the Clinical Specimens

Two hundred and sixty four clinical samples (196 NP swabs, 45 skin swabs, 15 serum, 7 sputum, 1 tracheal) were tested with the DNA assay. The results were shown in Table 4A. In this testing there were three indeterminants for FT and when reprobed as per standard protocol for indeterminants (OD of 0.300–0.399) they were found to be negative. There was one false positive (low positive) for FT (OD=0.838) which when retested was also found to be negative. The clinical specificity of the BioT DNA assay was 99.6% (263/264) (95% CI 98–100). Of the 549 clinical samples that were tested by the RNA assay, there was one false positive (OD=0.626) for HSN and when retested as per standard protocol for false positive (OD>0.400) it was found to be negative (OD=0.040). The overall clinical specificity of the BioT RNA assay was 99.8% (548/549) (95% CI 99–100).

### 2.4. Surrogate clinical testing

The results were shown in Table 4A. Surrogate clinical NP samples with FT (20/20), BA pX02 (20/20), YP (20/20), and skin specimens with VZV (20/20) and VM (20/20) were detected and had sensitivities of 100% (95% CI 83–100). BA pX01 was detected in 19/20 NP samples (sensitivity 95% (95% CI 75–100).

There was one sample positive for VZV in a clinical specimen spiked with VM. Using the standard assay protocol to re-probe any positive (∼30 minute procedure) it was found to be a true negative. This was most likely a technical error. However, even counting it as a false positive would not have much of an impact on the specificity for VZV which was 99.8% (95%CI 99–100). One sample tested very low positive for VM (OD=0.434) in the BA pX01 spiked samples. Re-probing of the PCR product continued to show the sample to be positive. However, retesting the original clinical sample demonstrated it to be negative. Therefore, this represented a true false positive (1/400 samples). Specificity for VM was 99.8% (95% CI 99–100). No other false positives where detected using the FT, BA pX01, BA pX02, and YP probes (specificities 100% (95% CI 99–100). Finally, all 36 negative control M4 samples were negative for all organisms.

A total of 104 serum specimens were used to test the surrogate clinical sensitivity and specificity of the RNA assay. The results are shown in Table 4B. For dengue 2, 3, and 4, 20/20 serum samples spiked were correctly detected resulting in sensitivities of 100% (95%CI 83–100), and dengue 1 testing showed 19/20 samples were correctly detected and the sensitivity was 95% (95% CI 75–100). No false positive results were found using any of the probes (including Ebola, HSN, RVF, and Lassa) and the BioT RNA assay showed 100% specificity (95% CI 96–100). The 24 negative control samples were negative for all targets making the overall specificity of the assay 100% (95% CI 99–100).

## 3. Discussion

The current study describes the development of two multiplex assays for detecting many CDC category “A” bioterrorism agents simultaneously. The first multiplex assay detects: *B. anthracis, F. tularensis, Y. pestis, V.major,* and Varicella zoster virus while the second multiplex assay detects: Ebola virus (Zaire), Lassa fever virus, Rift Valley fever virus, Hantavirus (Sin Nombre), and dengue virus (serotypes 1–4). Varicella-Zoster virus while not a category “A” agent was chosen as a target for the BioT DNA assay in order to positively identify a possible cause of rash or lesions in suspected smallpox patients in addition to demonstrating that a clinical sample was VM negative. It is presumed that virtually all of the patients coming in to a hospital emergency room with a concern for VM will not actually have this virus (short of a bioterrorism attack).

The virulence of *B. anthracis* has been associated with two mega plasmids, pX01 and pX02 [20]. Plasmid pX02 (60 MDa) carries the genes required for synthesis of an antiphagocytic poly-D-glutamic acid capsule facilitating host immune system evasion [20–25]. The 110-MDa plasmid pX01 is required for synthesis of the three anthrax toxin proteins, edema factor (EF), lethal factor (LF), and protective antigen (PA). These proteins act in binary combinations to produce the two anthrax toxins: edema toxin (PA and EF) and lethal toxin (PA and LF) [26]. Isolates lacking either the pX01 or pX02 plasmid are considered either avirulent or significantly attenuated [27,28]. In the present study, 2 sets of primers and probes were designed from the conserved regions of the *PA* and *Cap* gene located respectively on the plasmids pX01 and pX02 for *B. anthracis*. Only clinical samples that give positive signals for both primers and probe sets will be considered “positive” for BA. Since we demonstrated high sensitivity and specificity for both the pX01 and the pX02 primer and probe sets, our assay should work well for detecting both targets in BA.

The analytical testing of both multiplex assays demonstrated excellent LOD (1–10 input copies for all analytes) and no significant cross reactivity with a large number of agents that might be in clinical samples.

The clinical sample types used in the BioT DNA assay spiking experiments meet the CDC requirements for appropriate diagnostic samples for each Category “A” agent. CDC requires collection of blood, skin lesions or respiratory secretions for diagnosis of *B. anthracis*. Respiratory secretions and blood should be collected for diagnosis of *F. tularensis*, especially inhalational tularemia. Blood and lymphoid tissue specimens should be collected for diagnosis of *Y. pestis.* Bronchial/tracheal washing should be taken from suspected pneumonic plague patients. The skin of the vesicle top should be taken for diagnosis of VM. CDC prefers to collect blood or biopsy material of the lung and bone marrow aspirate for PCR diagnosis of VHF. After considering the CDC requirements, NP swab specimens were chosen for creating the surrogate “positive” clinical samples used in the spiking experiments for *B. anthracis*, *Y. pestis*, and *F. tularensis,* skin swab specimens were chosen for *V. major* and Varicella zoster virus, and serum specimens were chosen for the spiking experiments for dengue virus.

In the evaluation of the clinical specimens, 813 specimens in total were tested demonstrating very high sensitivity (in spiked samples) and specificity (spiked and unspiked samples) confirming that the assays are applicable to testing different clinical samples without inhibition or cross-reactivity and demonstrating their potential utility in clinical or public health use.

There are many molecular diagnostic methods that have been developed to detect bioterrorism agents [13,14,29]. Real-time PCR and micro-array are currently two of the most commonly reported molecular diagnostic methods. MGB probe-based real-time PCR assays were developed for detecting *Y. pestis, F. tularensis* and *B. anthracis* simultaneously [29]. For this test the sample must be tested simultaneously with a seperate singleplex assay for each of these three targets. Two commercial triplex (multiplex) real time PCR kits and machines were developed by Cepheid [13] and Idaho Technology, Inc. [14] to detect *B. anthracis, Y. pestis* and *F tularensis*, however, their assays can only be used on their machines. Therefore, laboratories with standard thermocyclers or real-time machines cannot run these assays. We were unable to purchase reagents for comparison testing. For viral hemorrhagic fever agents, several TaqMan or SYBR green real-time one-step RT-PCR assays have been developed for detecting and serotyping dengue [30–32]. A single-tube multiplex PCR was developed to identify the serotype of dengue virus which had been previously screened by real-time PCR [33]. The detection for Ebola virus, RVF and Lassa is still dependent on serology testing in most laboratories but additional RT-PCR methods have been recently developed. A German group established 6 one-step, real-time RT-PCR assays for Ebola virus, Marburg virus, Lassa virus, Crimean-Congo hemorrhagic fever virus, Rift Valley fever virus (RVFV), dengue virus (DENV), and yellow fever virus (YFV) [34]. These assays cannot distinguish all the targets simultaneously.

The BioT multiplex DNA and RNA assays are two new methods to compliment existing testing strategies. Possible advantages over published methods include increased number of analytes per test (5 *vs.* 1 or 3), stability of reagents, use of common technology and cost. Real-time reagents have shorter shelf lives than our HRP labeled reagents because of their use of fluorescent probes. In addition, our assay utilizes a standard PCR thermocycler and ELISA plate reader which cost tens of thousands of dollars less than real-time PCR machines and the reagent costs for our multiplex PCR-EHA format are less than real-time reagents. Additional side by side comparative research should be performed to assess the multiplex BioT DNA and RNA assays against other existing methods.

Both the BioT DNA assay and BioT RNA assay were developed using manual nucleic acid extraction (Roche). This method requires skilled technicians and is labor intensive. Recently there has been increased use of automated sample preparation using systems such as Roche’s MagnaPure Compact [35,36], the Qiagen BioRobot M48 [37,38] and Biomerieux’s NucliSens Extractor [39]. These robotic systems can dramatically increase throughput and decrease hands-on time. We recently demonstrated dramatic decreases in technician time (by half) using automated extraction and both EHA detection and automated microarray detection [40]. However, automation can have hidden variables that effect costs, sensitivity and reproducibility. The chemistry of each assay, including buffers and enzymes used, and especially the target (RNA *vs.* DNA and size) and clinical matrix can affect the results you obtain with some of the automated systems. We demonstrated with the BioT DNA assay that the sensitivity using Roche’s High Pure Viral Nucleic Acid Kit for extraction was one log better than using the Roche MagNAPure Compact system which also had less consistent reproducibility (data not shown). Ultimately, the goal of our laboratory is to develop fully contained, automated, hands-off devices that can extract nucleic acid, amplify a target, and detect it while being fast, accurate and inexpensive. This technology must be robust and flexible to different targets and matrix material (transport medium and clinical material). Medium to large multiplex assays as described in this paper are steps in this direction.

## 4. Materials and methodology

### 4.1. Primers and Probe Design

The primers and probes used in these 2 assays were designed from highly conserved regions of specific genes for each organism listed in Table 5A and 5B.

The primers and probe for the DNA assay specifically targeted the *Tul4* gene for *F. tularensis*, the *HA* gene for *V. major,* the *ORF29* gene for the Varicella zoster virus and the *Virulence Antigen* gene for *Y. pestis*. 2 pairs of primers for *B. anthracis* were designed from the conserved regions of the *Protective Antigen* (*PA)* and *Cap* gene located respectively on the plasmids pX01 and pX02 because both plasmids are required for pathogenicity. The primers and probes for the RNA assay specifically targeted the *L* gene for Ebola, the *GP2* gene for RVF, the *L* gene for the Lassa, the S segment for Hanta and the 3′UTR for dengue.

### 4.2. Bacterial Species and Genomic DNA

Varicella zoster virus (VZV) and all four serotypes of dengue virus were obtained from ATCC. Genomic DNA of FT and of BA (BA pX01-Sterne strain) were obtained from the Medical College of Wisconsin. Genomic DNA of YP was obtained from the Blood Center of Wisconsin. The genomic DNA of BA (BA pX02) was obtained from the University of Chicago.

### 4.3. Generation of Recombinant Positive Controls

To create positive controls, PCR products generated from whole organisms were purified and cloned into plasmids using the TOPO TA cloning system (Invitrogen, Carlsbad, CA), following the manufacturer’s instructions. All positive controls were sequenced to verify the amplicon sequences and the possibilities of any mismatches generated during cloning. The positive controls were further amplified to generate large quantities that were purified using Qiagen Plasmid Midi Kit (Qiagen, Valencia, CA). The synthetic RNA standards were generated from linearized target sequence plasmids by using T7 *in vitro* transcription system (Promega, Madison, WI).

The quantity of DNA and RNA transcripts were measured by the absorbance at 260 nm and the purity of the DNA or RNA by the ratio at A260/280. To determine the approximate copy number of plasmid controls and synthetic RNA controls, the absorbance value of the positive control (A_260_) was used in combination with Avogadro’s Number (6.022 × 10^23^) as described by Khanna *et al*. (2005) [41]. The RNA transcripts were diluted in RNase and DNase free water with RNase Inhibitor (Applied Biosystems, Foster City, CA), frozen at −80°C in 30 μL aliquots. Serial dilutions of the plasmids and RNA transcripts were used to establish the analytical sensitivity and specificity of BioT DNA and RNA multiplex assays.

### 4.4. The Standard Protocol of the BioT DNA and RNA Multiplex Assays

#### 4.4.1. PCR protocol for BioT DNA mPCR-EHA assay

Ten μL of extracted DNA were amplified in a 50 μL PCR reaction that contained 2.25 mM MgCl_2_ (Applied Biosystems), 0.8mM dNTP (Applied Biosystems), 1× PCR Buffer II (Applied Biosystems), 2.5 U of AmpliTaq Gold DNA polymerase (Applied Biosystems) and 6 pairs of primers at a final concentration 250nM for each forward primer and 500nM for each reverse primer. Amplification was performed in a GeneAmp 9700 thermocycler (Applied Biosystems). The PCR was carried out under the following conditions: 95° C for 10 min; 2 cycles of 95° C for 60 s, 55° C for 30 s and 72° C for 45 s; 38 cycles of 94° C for 60 s, 60° C for 30 s and 72° C for 30 s; and then an extension step at 72° C for 7 min.

#### 4.4.2. RT-PCR protocol of BioT RNA mRT-PCR-EHA assay

For the BioT RNA assay, a two-step RT-PCR was performed in a GeneAmp 9700 thermocycler (Applied Biosystems). cDNA was synthesized in a 20 uL reaction mixture containing 2.5 mM random hexamers (Applied Biosystems), 4 mM dNTPs (Applied Biosystems), 4mM MgCl_2_ (Applied Biosystems,), 1U/μL RNase inhibitor, 2.5U/μL MuLv reverse transcriptase (Applied Biosystems) and 3 μl RNA. The reaction mixture was incubated for 5 min at 25°C and for 30 min at 42°C and then the transcriptase was inactivated at 95°C for 5 min.

Next, 10 μL of cDNA were amplified by adding 39.5 μL of a Multiplex Supermix that contained 6 pairs of primers, appropriate buffers and 2.5 U of AmpliTaq Gold DNA polymerase (Applied Biosystems). The final concentration of primer is 250nM for each forward primer and 500nM for each reverse primer. Amplification was performed under the same conditions of the BioT DNA assay.

PCR products from both DNA and RNA assays were purified using the QiaQuick PCR purification Kit (Qiagen). A total of 65 μL of 10 nM horseradish peroxidase labeled probe solution (Eurogentec, San Diego, CA) was added to wells of a 96 well NeutrAvidin-coated microtiter plate (Pierce, Rockford, IL) and hybridized with 5 μL of the purified PCR product at 42°C for 30 minutes. The plate was washed 10 times with a 1X Wash Buffer. And then 200 μL of Tetramethyl benzidine substrate was added to each experimental well. The reaction was stopped by the addition of a stop solution (1N H_2_SO_4_) and the absorbance [optical density (OD)] was measured at 450 nm using a Molecular Devices spectrophotometer. The positive cut off value for this assay was chosen to be four times greater than the value of the negative control and greater than or equal to an OD value of 0.400 [15,16].

### 4.5. Analytical Sensitivity

The analytical sensitivity for the DNA assay was determined using serial dilutions of both DNA extracted from whole organisms and recombinant DNA controls. Ten-fold serial dilutions of genomic DNA (BA, YP, and FT), previously extracted from whole organisms and plasmid DNA controls were made (10^4^ to 10^1^ or 10^−1^ copies/mL) in M4 medium (Remel, Lenexa, KS), extracted using the High Pure Viral Nucleic Acid Kit according to manufacturer’s instructions (Roche Applied Science, Indianapolis, IN) and then tested. VZV whole organism was serially diluted (10^3^ to 10^−3^ CFU/mL) in M4 and extracted using the same method.

The analytical sensitivity for the RNA assay was determined using serial dilutions of both RNA transcripts and recombinant DNA controls for HSN, RVF, Lassa, and Ebola virus. The analytical sensitivity of the assay for dengue was determined using serial dilutions of all four serotypes of dengue virus. Ten-fold serial dilutions of RNA transcript and recombinant DNA control were made (10^7^ to 10^3^ copies/mL for RNA transcripts, 10^6^ to 10^2^ copies/mL for recombinant DNA control) using two matrices: M4 medium and RNase/DNase-free water. The RNA and plasmid controls diluted in water were used as a template in RT-PCR to determine the limit of detection (LOD) without extraction. The RNA controls diluted in M4 were extracted to determine the LOD with extraction. Dengue (4 serotypes) whole virus was serially diluted at 10^−2^ to 10^−5^ dilutions for dengue serotypes 1 and 2 (the virus stock for these serotypes came from ATCC without quantitation ), 10^5^ to 10^2^ LD_50_/mL for dengue serotype 3, and 10^3^ to 10^0^ LD_50_/mL for dengue serotype 4. Diluted whole viruses were extracted using high pure viral nucleic acid kit (Roche) according to manufacturer’s instructions and tested.

### 4.6. Analytical Specificity

Specificity of the DNA and RNA assays were evaluated by respectively testing 17 and 15 common respiratory pathogens that could be present in clinical specimens for potential cross-reactivity. ATCC bacterial and viral strains tested by the DNA assay were diluted to 1×10^3^ CFU/mL and 1×10^2^ −10^4^ TCID_50_ /mL. ATCC bacterial or viral strains tested by the RNA assay were diluted to 1×10^3^ CFU/mL, 1×10^4^ CFU/mL, 1×10^3^ TCID_50_ /mL or 1×10^4^ cells/mL respectively. The bacterial and viral strains were then tested using the same method as previously described. The plasmid DNA controls and whole virus, which are the targets of both BioT assays, were diluted and tested at 1 to 2 log higher than the LOD using the same method.

### 4.7. Evaluation of the Clinical Specimens

For the DNA assay, 264 clinical samples (196 NP swabs, 45 skin swabs, 15 serum, 7 sputum, 1 tracheal), were collected from 264 subjects at Children’s Hospital of Wisconsin and Froedtert Memorial Hospital during a 17-month period from October 2005 through March 2007 and were tested. Of the 264 subjects, 130 presented with respiratory symptoms and 134 presented without respiratory symptoms. Sixty three of the samples were children aged 0–6 years old, 20 were children aged 7–18 years old, 154 were aged 19–65 years old, and 27 were older than 66. Of the 264 clinical samples tested, 139 were females and 125 were males.

For the RNA assay, 549 clinical samples (417 NP swabs, 95 skin swabs, 21 blood, 12 sputum, 3 tracheal, 1 nasal swabs) were tested. The samples were collected from 418 subjects as described above. Of these samples, 302 were from the subjects with respiratory symptoms and 247 were from subjects that had no respiratory symptoms. Two hundred and thirty nine of the samples were from children age 0–6 years old, 106 were from children age 6–19 years old, 182 were from subjects age 19–65 years old and 22 were from subjects older than 66. Of the 549 clinical samples tested, 258 were from females and 291 were from males. Any sample with OD value lower than 0.300 was considered negative. Any sample with OD value between 0.300–0.399 was considered indeterminant and was reprobed to confirm the results. Any sample with OD value over 0.400 was considered positive and was retested.

### 4.8. Surrogate Positive Clinical Specimens

To evaluate the performance of the DNA assay we used surrogate positive clinical samples. A total of 120 clinical NP swab and skin swab specimens that previously tested negative with the same assay were used for spiking experiments. Twenty clinical specimens were spiked with plasmid DNA positive control and six M4 transport medium specimens were set up as negative specimens for each of the six organisms mentioned (36 total). The recombinant positive controls of FT and BA pX01 were spiked into NP swab specimens at the concentration 1×10^4^ copies/mL. The recombinant positive controls of YP and BA pX02 were spiked into NP swab specimens at the concentration 1×10^5^ copies/mL. The recombinant positive control of VM was spiked into skin swab samples at the concentration 1×10^4^ copies/mL. The VZV whole organism was spiked into skin swab specimens at 1×10^0^ TCID_50_/mL.

To evaluate the performance of the RNA assay for positive clinical samples, pooled serum samples that previously tested negative with this assay were aliquoted into 400μL samples and used for the spiking experiment. Twenty serum samples were spiked with whole organism of each of the serotypes of dengue virus (80 total) and 6 serum samples were run as negative specimens with each serotype of dengue virus (24 total). Dengue 1 and dengue 2 were spiked at 1×10^−2^ dilution, the concentration of which was 2 logs higher than the LOD of dengue 1 and 1 log higher than the LOD of dengue 2. Dengue 3 was spiked at 5×10^5^ LD_50_/mL, the concentration of which was 1.5 logs higher than the LOD of dengue 3. Dengue 4 was spiked at 1×10^4^ LD_50_/mL, the concentration of which was 2 logs higher than the LOD of dengue 4. All of these spiked specimens were tested blindly.

## 5. Conclusion

The presented data demonstrates that the multiplex BioT DNA assay and BioT RNA assay are highly sensitive, specific and reproducible in testing genomic DNA, recombinant plasmid positive control, RNA transcription, whole organism and spiked clinical specimens utilizing a standard thermocycler. The assays are also rapid (∼5 hrs), utilizes relatively inexpensive reagents (∼$50 for 1 sample which was tested for all target analytes, ∼$10/analyte) and requires little technician time per sample (∼3 hrs). Compared to other currently available assays for laboratory diagnosis of bioterrorism agents, the BioT assays represents useful new tools for clinicians and public health officials who need to detect these important pathogens in people either from natural infection or man-made exposure (bio-terrorism).

## Figures and Tables

**Table 1.** Limits of detection for BioT DNA assay using genomic DNA and plasmid controls for each organism.

**Organism**	**Genomic DNA LOD**	**Plasmid Control LOD**
BA pX01	1 × 10^1^ copies/mL	1 × 10^2^ copies/mL
BA pX02	1 × 10^2^ copies/mL	1 × 10^2^ copies/mL
FT	1 × 10^1^ copies/mL	1 × 10^3^ copies/mL
YP	1 × 10^0^ copies/mL	2.5 × 10^2^ copies/mL
VZV	1 × 10^−2^ TCID_50_/mL	ND
VM	ND	5 × 10^2^ copies/mL

ND - The LOD of genomic DNA for *Variola major* wasn’t tested because the genomic DNA of VM was not available and no control for VZV was tested since whole virus is readily available.

**Table 2.** The limits of detection of the BioT RNA assay for RNA transcript control, plasmid controls, or whole virus.

Organism	Without Extraction				With Extraction	
	RNA Controls		Plasmid Controls		RNA Controls/Whole Virus	
	Copies/mL	Copies/rxn	Copies/mL	Copies/rxn	Concentration	Copies/rxn
Ebola	1 × 10^6^	1500	1 × 10^3^	1.5	1 × 10^5^ copies/mL	1200
RVF	1 × 10^6^	1500	1 × 10^3^	1.5	1 × 10^6^ copies/mL	12000
Lassa	1 × 10^5^	150	1 × 10^3^	1.5	1 × 10^5^ copies/mL	1200
Hanta SN	1 × 10^4^	15	1 × 10^3^	1.5	1 × 10^5^ copies/mL	1200
Dengue 1					1 × 10^−4^ dilution	
Dengue 2					1 × 10^−3∼−4^ dilution	
Dengue 3					1 × 10^4–5^ LD_50_/mL	
Dengue 4					1 × 10^2^ LD_50_/mL	

**Table 3.** Specificity of the BioT DNA and BioT RNA assays was tested against different pathogens.

**A.** The organism, dilution and the result of the specificity testing for BioT DNA multiplex assay.						
Organisms	Concentration of strains	Target				
		*B. anthracis*	*F. tularenisis*	V. major	*Y. pestis*	VZV
Adenovirus C	10^3^ TCID_50_/mL	Neg	Neg	Neg	Neg	Neg
Influenza A	10^3^ TCID_50_/mL	Neg	Neg	Neg	Neg	Neg
*Francisella philomiragia*	10^−4^dilution	Neg	Neg	Neg	Neg	Neg
*Bacillus cereus*	10^−4^dilution	Neg	Neg	Neg	Neg	Neg
*Bacillus thuringiensis*	10^−4^dilution	Neg	Neg	Neg	Neg	Neg
*Yersinia pseudotuberculosis*	10^−4^dilution	Neg	Neg	Neg	Neg	Neg
*Yersinia enterocolitica*	10^−4^dilution	Neg	Neg	Neg	Neg	Neg
*Clostridium perfringens*		Neg	Neg	Neg	Neg	Neg
Vaccinia Virus		Neg	Neg	Neg	Neg	Neg
Epstein-Barr virus	10^2^ TCID_50_/mL	Neg	Neg	Neg	Neg	Neg
Cytomegalovirus	10^4^ TCID_50_ /mL	Neg	Neg	Neg	Neg	Neg
*Mycoplasma pneumoniae*	10^−3^dilution	Neg	Neg	Neg	Neg	Neg
*Chlamydia pneumoniae*		Neg	Neg	Neg	Neg	Neg
*Legionella pneumophila*		Neg	Neg	Neg	Neg	Neg
*Mycoplasma pneumoniae*	10^−3^dilution	Neg	Neg	Neg	Neg	Neg
Human metapneumovirus	10^4^ TCID_50_/mL	Neg	Neg	Neg	Neg	Neg
Respiratory syncytial virus A	10^3^ TCID_50_/mL	Neg	Neg	Neg	Neg	Neg
*Bacillus anthracis*	10^−4^dilution	>3.0	Neg	Neg	Neg	Neg
*Francisella tularensis*	10^4^ CFU/mL	Neg	>3.0	Neg	Neg	Neg
*Variola major*	10^−4^dilution	Neg	Neg	>3.0	Neg	Neg
*Yersinia pestis*	10^−4^dilution	Neg	Neg	Neg	>3.0	Neg
Varicella-Zoster virus	10^3^ TCID_50_/mL	Neg	Neg	Neg	Neg	>3.0
Negative control		Neg	Neg	Neg	Neg	Neg

**B.** The organism, dilution and the result of the specificity testing for BioT RNA multiplex assay.							
Organisms	Concentration of strains	Target					
		Ebola	Hanta	Lassa	RVF	Dengue	IC
Human parainfluenza viruses 1	10^3^TCID_50_ /mL	Neg	Neg	Neg	Neg	Neg	Neg
Human parainfluenza viruses 2	10^3^TCID_50_ /mL	Neg	Neg	Neg	Neg	Neg	0.957
Human parainfluenza viruses 3	10^3^TCID_50_ /mL	Neg	Neg	Neg	Neg	Neg	Neg
Influenza A	10^3^TCID_50_ /mL	Neg	Neg	Neg	Neg	Neg	Neg
*Mycoplasma pneumoniae*	10^4^ cells /mL	Neg	Neg	Neg	Neg	Neg	Neg
Respiratory syncytial virus A	10^3^TCID_50_ /mL	Neg	Neg	Neg	Neg	Neg	Neg
Respiratory syncytial virus B	10^3^TCID_50_ /mL	Neg	Neg	Neg	Neg	Neg	Neg
Varicella-Zoster virus	10^3^TCID_50_ /mL	Neg	Neg	Neg	Neg	Neg	Neg
*Human Coronavirus OC43*	10^3^TCID_50_ /mL	Neg	Neg	Neg	Neg	Neg	Neg
Enterovirus type 71	10^3^TCID_5_0 /mL	Neg	Neg	Neg	Neg	Neg	Neg
Human Coronavirus 229E	10^3^TCID_50_ /mL	Neg	Neg	Neg	Neg	Neg	Neg
Adenovirus type 3	10^3^TCID_50_ /mL	Neg	Neg	Neg	Neg	Neg	Neg
*Staph pneumoniae*	10^4^ cfu /mL	Neg	Neg	Neg	Neg	Neg	Neg
*Staph aureus*	10^3^ cfu /mL	Neg	Neg	Neg	Neg	Neg	Neg
*Legionella micdadei*	10^4^ cells /mL	Neg	Neg	Neg	Neg	Neg	Neg
Ebola	10^4^ copies/ml	1.786	Neg	Neg	Neg	Neg	Neg
Hanta	10^4^ copies/ml	Neg	3.796	Neg	Neg	Neg	Neg
Lassa	10^4^ copies/ml	Neg	Neg	2.651	Neg	Neg	Neg
RVF	10^4^ copies/ml	Neg	Neg	Neg	3.259	Neg	Neg
Dengue 1	10^−2^ dilution	Neg	Neg	Neg	Neg	2.282	Neg
Dengue 2	10^−2^ dilution	Neg	Neg	Neg	Neg	3.943	Neg
Dengue 3	5x10^5^TCID_50_ /ml	Neg	Neg	Neg	Neg	4.000	Neg
Dengue 4	10^4^TCID_50_ /mL	Neg	Neg	Neg	Neg	3.249	Neg
Negative control		Neg	Neg	Neg	Neg	Neg	Neg
Detection control		3.502	3.980	2.454	3.903	3.876	4.000

Neg = negative. Optical density readings less than 0.400 are indicated by negative.

The positive OD reading is the average OD reading of duplicate samples.

**Table 4.** The sensitivity and specificity of BioT DNA and BioT RNA assays in spiked clinical specimens.

**A.** Sensitivity and specificity of BioT DNA assay.						
Organism	*Francisella tularensis*	*Bacillus anthracis* (pX01)	*Bacillus anthracis* (pX02)	*Yersinia pestis*	VZV	*Variola major*
Detail						
Spiking Concentration	1×10^4^ copies/ml	1×10^4^ copies/ml	1×10^5^ copies/ml	1×10^5^ copies/ml	1×10^0^ TCID50/ml	1×10^4^ copies/ml
OD(mean±SD)	2.643±0.843	2.727±1.294	3.289±0.289	2.638±0.458	3.968±0.074	3.564±0.464
Sensitivity (95%CI)	100%(20/20) (83–100%)	95%(19/20) (75–100%)	100%(20/20)	100%(20/20)	100%(20/20)	100%(20/20)
Negative Controls	(-)	(-)	(-)	(-)	(-)	(-)
M4 NEG controls	6/6	6/6	6/6	6/6	6/6	6/6
False Positive		0.434 VM (re-amp -)				0.733 VZV (re-probe -)
This experiment Specificity, clinical (95%CI)	100%(100/100) (96–100%)	100%(100/100)	100%(100/100)	100%(100/100)	99%(99/100) (95–100%)	99%(99/100)
Specificity-Controls (95%CI)	100%(36/36) (90–100%)	100%(36/36)	100%(36/36)	100%(36/36)	100%(36/36)	100%(36/36)
Specificity, overall (95%CI)	100% (400/400) (99–100%)	100%(400/400)	100%(400/400)	100%(400/400)	99.8%(399/400) (99–100%)	99.8%(399/400)

**B.** Sensitivity and specificity of surrogate testing for BioT RNA assay.				
Organism	Dengue 1	Dengue 2	Dengue 3	Dengue 4
Detail				
Spiking Concentration	1×10^−2^ dilution	1×10^−2^ dilution	5×10^5^ LD_50_/mL	1×10^4^ LD_50_/mL
OD(mean±SD)	2.486±1.305	1.077±0.373	1.248±0.147	1.462±0.517
Sensitivity (95%CI)	95%(19/20) (76.2–98.8%)	95%(20/20) (83.9–99.9%)	100%(20/20) (83.9–99.9%)	100%(20/20) (83.9–99.9%)
Negative Controls	(-)	(-)	(-)	(-)
NEG sample	6/6	6/6	6/6	6/6
This experiment Specificity (95%CI)	For Ebola 100% (104/104) (96–100%)	For Lassa 100% (104/104) (96–100%)	For RVF 100% (104/104) (96–100%)	For HSN 100% (104/104) (96–100%)

**Table 5A:** Primer and probe sequence using in the BioT DNA multiplex PCR-EHA assay.

Organisms	Primers & probes	Gene	Size of Amplicon	Sequences
*Bacillus anthracis*	BA pX01-1F	PA (pX01)	311 bp	5′-ggatttcaagttgtactggaccgat-3′
	BA pX01-1R			5′-ctgtacggatcagaagccgtgctcca-3′
	BA pX01-P			5′-ctagtgataacttacaattgccagaat-3′’
	BA pX02-1F	Cap (pX02)	305 bp	5′-tgtccattatatggaatggtagcagtg-3′
	BA pX02-1R			5′-tggtacatctgcgcgaatgatatattggt-3′
	BA pX02-P			5′-acattcacaaataagtgcttctgcttc-3′
*Francisella tularensis*	FT-1F	Tul 4	156 bp	5′-ataacccaccaaggaagtgtaagat-3′
	FT-1R			5′-cacttaccgctacagaagttatta-3′
	FT-P			5′-aggctccagaaggttctaagtgccatgata-3′
*Yersinia pestis*	YP-1F	VA	195 bp	5′-cggaggtttttgccaataga-3′
	YP-1R			5′-actgccatgaacgcccgcaattc-3′
	YP-P			5′-tgccattcttaaaggcggtcatta-3′
*Variola major*	VM-1F	HA	124 bp	5′-cacaacagacaagacgtccg-3′
	VM-1R			5′-catcattggcggttgattta-3′
	VM-P			5′-acgtcgggaccaattactaataaaga-3′
*Varicella Zoster virus*	VZV-1F	ORF29	226 bp	5′-gctgacacagccttgcacgcagaag-3′
	VZV-1R			5′-tcggtcatcccgctatcctccacctcag-3′
	VZV-P			5′-caacactggaatttacgaagaaactccaacagatatc-3′

**Table 5B:** Primer and probe sequence using in the BioT RNA multiplex RT-PCR-EHA assay.

Organisms	Primers & probes*	Gene	Size of Amplicon	Sequences
Ebola virus (Zaire)	Ebola-1F	L	243 bp	5′gatgcagtattcgagcctaatgttctag-3′
	Ebola-1R			5′-gtgtttgaacattgcgagtcggataag-3′
	Ebola-P			5′-actcgagtatctactaccacaatatcggaac-3′
Lassa fever virus	Lassa-1F	L	197 bp	5′-agcctgatcccagatgccacacatctag-3′
	Lassa-1R			5′-tgctgttggagcggctgatggtctcag-3′
	Lassa-P			5′-gcctggttgagtgcaacaaccactatctgtgtctcaactg-3′
Rift Valley fever virus	RVF-1F	GP2	196 bp	5′-gacgcagcattttgctctgcttatg-3′
	RVF-1R			5′-gttgtgcaaggctcaactctctggatg-3′
	RVF-P			5′-ctttatgtgtagggtatgagagagtggttgtga-3′
Hantavirus (Sin nombre)	Hanta-1F	S segment	222 bp	5′-gcaccctcaaagaagtgcaagacaaca-3′
	Hanta-1R			5′-gaagccaatttctgagctgcaata-3′
	Hanta-P			5′-gctgtgtctgcattggaiaccaaactcg-3″
Dengue virus	Dengue-2F	3′UTR	141 bp 225 bp	5′-aaggactagiggttakaggagacc-3′
	Dengue-2R A Dengur-2R B			5′-ctgttgattcaacagcaccattc-3′ 5′-ctgttggatcaacaacaccaatc-3′
	Dengue-P			5′-aacagcatattgacgctgggaiagaccaga-3′

* F is forward primer, R is reverse primer.
